# A Bitter Experience That Enlightens the Future: COVID-19 Neurological Affection and Perspectives on the Orexigenic System

**DOI:** 10.7759/cureus.30788

**Published:** 2022-10-28

**Authors:** Sherine Abdelmissih

**Affiliations:** 1 Medical Pharmacology, Kasr Al-Ainy Faculty of Medicine, Cairo University, Cairo, EGY

**Keywords:** oxs, orexigenic system, neurological disorders, stress, diabetes mellitus, obesity, inflammation, angii, ace2, covid-19-retro

## Abstract

The history of coronaviruses revealed that these viruses caused multiple outbreaks in the past, including a previous severe acute respiratory syndrome (SARS) outbreak in 2003. In 2019, a novel SARS virus, SARS-CoV-2, started a drastic pandemic that, up till now, keeps peaking in successive waves owing to the mutational ability of the virus versus the short-term immunity against it. Although the angiotensin-converting enzyme 2 (ACE2) is the gate through which the virus gets access to human cells, yet ACE2 is deemed protective in lung injury yielding vasodilator, anti-fibrotic, and anti-inflammatory peptides. The viral-provoked ACE2 downregulation aggravated a subsequent potentially lethal cytokine storm. Both the tumor necrosis factor-alpha (TNF-α) receptor (TNFR), activated by the proinflammatory cytokine, TNF-α, released during coronavirus disease 2019 (COVID-19), and ACE2 are cleaved by tumor necrosis convertase enzyme (TACE) to render respective soluble decoy mediators. Several risk factors were linked to COVID-19 morbidity and neurological affection, including obesity and diabetes mellitus (DM), attributed to ACE2 overexpression in obesity, a low-grade inflammatory state with both obesity and DM, and defective lung reparative machinery, added to low tissue-to-lung ACE2 expression in DM. The ACE2 shedding by SARS-CoV-2 upon its entry into the brain, together with the inflammatory cytokines invading the brain, predispose to such neurological affection. However, ACE2 was not sufficient to justify the occurrence of neurological disorders with COVID-19, owing to its lower brain expression, relative to other tissues. Other mediators should have contributed to such neurological disorders, of which, orexins (OXs) are discussed, owing to multiple functional similarities to ACE2. Eventually, this review highlights such similarities selected according to their possible relevance to COVID-19 symptomatology and pathology. Both ACE2 and OXs confer anti-inflammatory benefits, reduce cerebral endothelial dysfunction, promote neuronal survival and neurogenesis, and add to their therapeutic potentiality in sepsis. Both ACE2 and OXs assist in moderating the stress responses and the stress-activated hypothalamic-pituitary-adrenal axis. Both ACE2 and OXs are affected by obesity and DM. The loss of ACE2 and OXs signaling was suggested in neuro-inflammatory and neurodegenerative diseases. Of interest is the abundance of OXs in the dissemination routes to the brain, namely, the peripheral olfactory and the enteric systems. The presumptive role of OXs as analgesics and antipyretics might add to their favorable profile. Advantageously, the availability of OXs agonists and antagonists makes it applicable to corroborate or abrogate the future utility of targeting the orexigenic system in terms of COVID-19 neurological affection. Elaborative work, exploring in vitro and in vivo models, is recommended to identify or deny such perspective involvement.

## Introduction and background

The coronavirus disease 2019 (COVID-19) pandemic started in late 2019, followed by successive waves of infection that vary in severity, symptomatology, and causative viral variants. The novel coronavirus attacks the respiratory passages in mammals to produce mild to severe acute respiratory syndrome, hence its name, SARS-CoV-2, associated with uncontrolled inflammatory response or the cytokine storm [1].

The 2019 coronavirus is a member of the coronaviruses (*Coronaviridae* family); it is known to be transmitted among humans as an airborne infection. It replicates in the ciliated epithelium of the nasal mucosa, the first site to suffer inflammation, then proceeds to flu-like symptoms that end either as a self-limiting disease or gradually progressing to deadly lung fibrosis or disseminated inflammatory crisis (cytokine storm). Unfortunately, the immunity against coronaviruses is short-lived, lasting only one to two years, indicating that re-infection can occur and recur [2], which was the case over the past years.

Anticipating that pandemic-triggered stress rationalizes the prevalence of anxiety, depression, and panic disorders, among other stress-related neuropsychiatric disorders, whether in the active disease stage [3] or during recovery [4], does not mean that direct brain invasion is exempted. The central viral dissemination is an issue keeping researchers at the pace to explore the novel presumptive linkage between the virus and the brain neural circuits. Despite multiple studies and reviews tracking, proficiently, such COVID-19-induced neurological affection [5-8], knowledge gaps still exist about the machinery and management of such viral-to-brain accessibility and localization.

Taken the lower brain expression of angiotensin-converting enzyme 2 (ACE2), relative to other tissues, ACE2, alone, cannot verify the incidence of neurological affection observed with COVID-19, and other mediators should be considered, of which orexins (OXs) were chosen based on multiple functional similarities with ACE2 and their implications in various neurological disorders that can be triggered by SARS-CoV-2 as well. Such orexigenic neurologic involvement was also verified by in vitro and in vivo experimental models as well as human studies. The wide brain distribution of OXs and their abundance along the olfactory and enteric routes facilitating viral brain invasion are noteworthy. Above all reasons, existing pharmacological agents that modify OXs signaling endorsed tracking this system as a prospective target in the COVID-19 pandemic. Eventually, this review highlighted these aspects, which are of potential relevance to COVID-19.

## Review

Severe acute respiratory syndrome coronavirus 2 (SARS-CoV-2), a member of the Coronaviridae family

A historical markup of coronaviruses revealed that they have been implicated in zoonotic diseases, such as the severe acute respiratory syndrome coronavirus (SARS-CoV) transmitted from civets (cat-like creatures) [9], which was responsible for an outbreak dating back to 2002-2003 [10], the Middle East respiratory syndrome coronavirus (MERS-CoV) transmitted from camels [11], as well as swine acute diarrhea syndrome coronavirus (SADS-CoV) transmitted from pigs [12].

The coronaviruses family comprises enveloped single-stranded (positive-sense) ribonucleic acid (RNA), provided with nucleoprotein in a capsid of matrix protein. The novel coronavirus is one of the largest among RNA viruses [13]. Once inside the host cell, the uncoated genome is transcribed and translated into multiple assemblies of mRNAs [14]. The RNA genome of SARS-CoV-2 encodes four structural proteins (S, E, M, N) in 5’-3’ order, namely, spike (S) glycoprotein, club-shaped projections giving these pathogens the characteristic crown (corona) appearance [2], envelope (E) protein, matrix (M) protein as the most abundant, spanning viral envelope [15], and nucleocapsid (N) protein [16].

One of the major differences between cognate species relies on the portal of entry. The novel coronavirus infects human cells by targeting ACE2, facilitated by the cellular transmembrane serine protease-2 (TMPRSS2), which primes the spike-protein (Sp) of the virus to attach to ACE2 [17]. This viral/enzyme binding yields conformational changes of ACE2, causing either destruction of the ectodomain together with endocytosis of the transmembrane domain or internalization of the whole enzyme as such [18], together with the attached virus. In vitro human airway epithelial cells studies and acid-induced lung injury in mice revealed that, upon the sequestration of ACE2 from the cell membrane, affected cells suffered a more aggressive phenotype [17].

Interestingly, the novel coronavirus exceeded the former SARS-CoV, binding affinity to human ACE2 by 10- to 20-fold, hence its fast and furious transmission among humans [19], as was detected in mice model of acute lung injury (ALI) upon injecting the viral Sp [20]. Currently, no evidence of SARS-CoV-2 binding to angiotensin-converting enzyme (ACE) is present [21], despite 60% similarity with ACE2 genomic structure [10]. Both ACE and ACE2 are part of the renin-angiotensin system (RAS). The SARS-CoV-2/ACE2 complex activates RAS, by causing direct loss of ACE2, leading to the defective breakdown of angiotensin II (ANGII) [22].

ACE2 and involvement in lung injury

Although several decades have elaborated since the identification of the RAS, every now and then, this system emerges as the secret key that discloses interrelated pathways, relevant to human health and disease. It is one system with pleiotropic effects on cardiovascular, endocrinal, renal, musculoskeletal, respiratory, and neurological systems.

**Figure 1:**
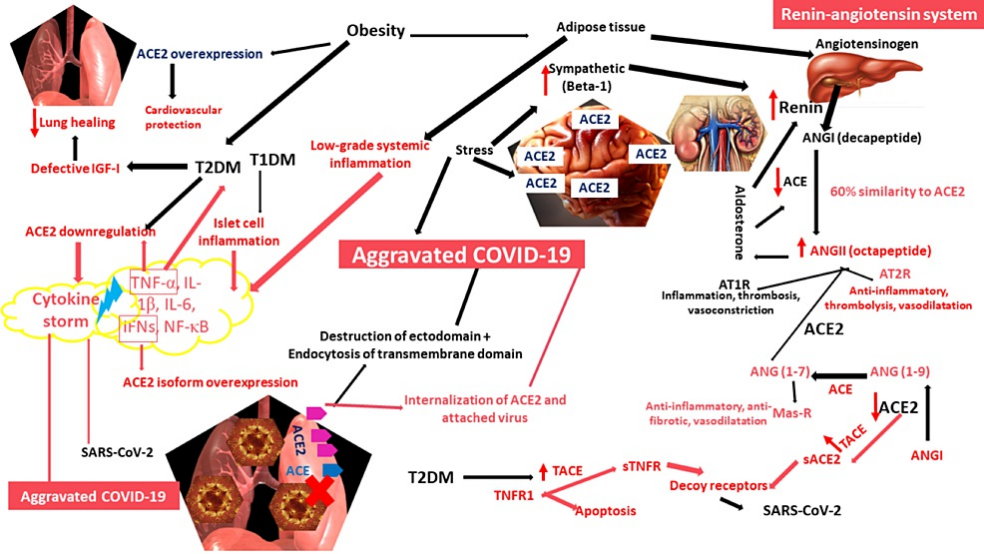
COVID-19, renin-angiotensin system, and the risk with obesity, diabetes mellitus, and stress. The RAS consists of renin, yielding ANGI from angiotensinogen; ANGI is converted by ACE2 to ANG (1-9) and by ACE to ANGII. Effects of ANGII binding to AT1R overwhelm the AT2R binding effects. ACE and ACE2 convert ANG (1-9) and ANGII, respectively, into ANG (1-7) that exerts similar functions to AT2R upon binding to Mas-R. Sympathetic stimulation, during stress, increases renin release and stress-related responses, owing to abundant brain ACE2. Stress is related to aggravated COVID-19. The binding of SARS-CoV-2 and the cytokine storm elicit ACE2 downregulation and an aggravated COVID-19 phenotype. In obesity, ACE2 overexpression, albeit conferring cardiovascular protection, increases the likelihood of viral attachment and invasion of the lungs, adding to a low-grade systemic inflammation triggered by excess adipose tissue. Increased release of angiotensinogen via adipose tissue. Obesity is a predisposing factor to T2DM. In T1DM, islet cell inflammation exacerbates the viral cytokine storm. In T2DM, ACE2 is downregulated, and the viral cytokine storm further aggravates both. The overexpression of TACE in T2DM increases the formation of ACE2 and TNFR1, acting as decoy receptors for SARS-CoV-2. COVID-19: coronavirus disease 2019; RAS: renin-angiotensin system; ANGI: angiotensin I; ACE: angiotensin-converting enzyme; ANGII: angiotensin II; ACE2: angiotensin-converting enzyme 2; sACE2: soluble angiotensin-converting enzyme 2; AT1R: angiotensin receptor-1; AT2R: angiotensin receptor 2; Mas-R: Mas receptor; ANG (1-7): angiotensin (1-7); ANG (1-9): angiotensin (1-9); TACE: tumor necrosis factor/angiotensin convertase; T1DM: type 1 diabetes mellitus; T2DM: type 2 diabetes mellitus; IGF-I: insulin-like growth factor-I; TNF-α: tumor necrosis factor-alpha; IFNs: interferons; IL-1β: interleukin-1beta; IL-6: interleukin-6; NF-κB: nuclear factor-kappa B; TNFR1: tumor necrosis factor-alpha receptor 1; sTNFR: soluble tumor necrosis factor-alpha receptor.

The release, synthesis, and functions of RAS components as well as the placement of ACE2 in this pathway [23-26] are depicted in (Figure 1).

In healthy adults, the vasodilator, anti-inflammatory angiotensin receptor-2 (AT2R) expression is too low to counteract angiotensin receptor-1 (AT1R) dynamics [27]. ACE2 is found in the pulmonary epithelium, specifically on type II alveolar cells and lymphocytes [28]. Considering that ACE2 showed preferential localization in the surfactant-producing type II alveolar cells, as well as the respiratory ciliated epithelial cells, patients with COVID-19 and inflammatory lung injury will exhibit reduced surfactant and susceptibility for lung collapse, justifying the respiratory deterioration in severe cases [29]. Like the low AT2R expression in healthy adults, ACE2 might be insufficient to antagonize the ANGII effects, even with a compensatory increase in disease states [30]. Furthermore, the pharmacologic RAS blockade interrupts the negative feedback loop exerted by ANGII on renin, resulting in a hyperreninemic state. In turn, renin can exploit non-ACE pathways to generate ANGII, increasing the vasoactive angiotensin subtypes upstream to RAS blockade such as angiotensin III (ANGIII) [31].

In children with respiratory syncytial virus (RSV), increased ANGII, as a consequence of ACE2 disruption, was observed [26,32], thus, Gu et al. [33] exploited this finding to manage the infected mouse model of acute respiratory distress syndrome (ARDS) with recombinant ACE2, yielding promising cytoprotective effect against ALI. On the same token, influenza virus strains (H5N1 and H7N9) were found to upregulate ANGII, with subsequent AT1R stimulation, while downregulating ACE2, and altogether they were suggested as facilitators of lung injury [34]. When other components of the RAS system were measured in the early course of ARDS, ANGI levels were high in lethal situations and low in survivors who also had even higher Ang1-9 and Ang1-7, suggesting a plausible compensatory increased ACE2 activity [35]. Recently, an ACE2 isoform overexpression in respiratory ciliated epithelial cells was induced by interferons (IFNs) and RNA respiratory viruses [36].

The cytokine storm of COVID-19: ACE2 as linked to tumor necrosis factor-alpha

As a known frenemy of the human body, the immune system recruitment, elicited by viruses, will generate pro-inflammatory cytokines, such as tumor necrosis factor-alpha (TNF-α), nuclear factor-kappa B (NF-κB), IFNs, and interleukins (ILs), added to various immune cells, such as the natural killer cells, neutrophils, and monocytes, aided by the special forces of T- and B-lymphocytes. Such defensive immune mobilization will generate neutralizing antibodies and supposedly long-lasting memory cells [37]. Unfortunately, the relevant memory cells to SARS-CoV-2 had a three-month average life span [38], which could not sustain the speedy mutating virus, resulting in recurrent infections. Furthermore, the viral inflammatory response could be aggravated with disequilibrated high ACE/ANGII/AT1 against low ACE2/angiotensin7-9/AT2 and Mas [39], eventually culminating in drastic tissue damage. The cytokine storm, the state of pro-inflammatory cytokines overproduction, corresponded to the COVID-19 adverse outcomes [40], as was seen when IL-6 was correlated to mortality and the need for ventilators in patients with COVID-19 [41].

The cell surface TNF-α is shedded by the activity of tumor necrosis factor convertase (TACE) or a member of the adamalysin enzymes (ADAM17) [42], to yield soluble TNF-α (sTNF-α). Within limits, sTNF-α helps activate the innate immune system; but exceeding the limits can result in autoimmune diseases such as rheumatoid arthritis and Crohn’s disease [43]. By acting on TNF receptor-1 (TNFR1), the sTNF-α level was correlated to the degree of lung fibrosis in idiopathic pulmonary fibrosis (IPF) [44]. TNF-α is not the only ligand for TACE, as both TNFR1 and TNF receptor-2 (TNFR2) are also substrates [45], as well as ACE2, the TACE-mediated proteolysis of which is enhanced by ANGII (Figure 1) [46]. Concomitant downregulation of ACE2 is associated with virus-induced overexpression of TACE to mediate more shedding of the residual cell membrane-anchored ACE2 [47], enhancing the availability of sTNF-α [48], which, in turn, exacerbates ACE2 downregulation and TACE upregulation, so that a vicious circle is generated until no more players exist to complete the round. Soluble forms of ACE2 and TNFR might act as decoy receptors for SARS-CoV-2 [49] and other viruses, including poxviruses [50], to which the monkeypox virus belongs. Luckily, soluble ACE2 and TNFR conveyed some protection from brain stroke [51].

Epidemiology of COVID-19

Owing to the fast-track COVID-19 strain mutations, epidemiological studies, in 2022, were released by the World Health Organization (WHO) [52] on weekly basis. A quick overview from March 2022 to July 2022 revealed that the pattern of dissemination has shifted from an overt decline over several weeks to a noticeable rise. In the United Kingdom, in July 2022, one in every 20 people was positive for COVID-19 based on nose and throat swabs analysis of various omicron subvariants. Fortunately, a regressing pattern was reported as well [53].

In 2022, the Centers for Disease Control and Prevention (CDC) [54] declared that the largest cumulative number of cases was accredited to young adults (18-29 years old), yet with higher rates of hospitalization and mortality among those aged 85 years and older. A meta-analysis of systematic reviews, including 57 studies, declared that men are more affected than women, mostly due to gender-specific differences in smoking and alcohol consumption, added to being more active outside their homes whether at work or in leisure time [55]. This was also valid referring to the Global Health 5050 data disclosing that confirmed cases and death rates were higher in men in different countries [56].

Epidemiological studies identified various comorbidities that pose a high risk for COVID-19-related health hazards, including obesity and diabetes mellitus (DM) [57], possibly attributed to the downregulation of ACE2 in DM [58], which is exacerbated by viral binding, and the inflammatory and fibrotic responses induced by TNF-α and transforming growth factor-beta (TGF-β) [59].

The prevalence of anxiety, depression, and post-traumatic stress disorder (PTSD) was high following critical illnesses in survivors of COVID-19 [60-62]. In a tertiary hospital, around 60% of cases with COVID-19-related neurological complications had DM, 58% were hypertensive, and 9% were obese [63]. The aggressive phenotype of COVID-19 in patients with DM was attributed to an exaggerated inflammatory response, immunocompromization, lung injury, and increased infectivity of the virus, especially with co-existing obesity, as well as other comorbidities [64].

Obesity and diabetes mellitus as predisposing to COVID-19

Obesity is considered one of the risk factors predisposing to COVID-19 morbidity, especially when the body mass index (BMI) exceeds 30 kg/m2 [28]. In high-fat, obese, male wild-type mice, higher ACE2 and TMPRSS2 expressions were detected in the lungs, even exceeding pulmonary TMPRSS2 expression in female obese mice [65], providing an enriched field for the virus to load. In obese individuals, the ACE2 upregulation was regarded as a compensatory mechanism to counteract the factors prompting other comorbidities such as hypertension and cardiac injury [66,67]. Obesity itself is a triggering factor when local inflammatory response starts in adipose tissue, engendering a systemic low-grade inflammatory state [68]. Special consideration was given to the involvement of TNF-α, IL-6, and NF-κB, among others, in both the obesity-provoked inflammation and the development of DM [69]. Furthermore, obesity is one of the well-established predisposing factors to the development of insulin resistance and type 2 DM (T2DM) [70].

The COVID-19 course seems more severe and more prolonged in patients with DM [58]. A high fasting blood glucose in patients with COVID-19 correlated to a worse COVID-19 prognosis and higher mortality [71]. In type 1 DM (T1DM), macrophages releasing TNF-α and IL-1 trigger beta-islet cell inflammation [72], substantiated by abundant lymphocytes and neutrophils from the surrounding pancreatic exocrine part [73], while in T2DM, obesity, a known risk factor for COVID-19, is a strong determinator of reduced insulin sensitivity [74]. The inflammatory theory of T2DM is likely adopted, especially that the level of inflammatory markers could predict insulin resistance and the development of T2DM [75]. If DM is an inflammatory disease, this would corroborate the promotion of hypercytokinemia (cytokine storm) upon invasion of SARS-CoV-2 in such a susceptible subset of patients.

In the respiratory setting, insulin-like growth factor-I (IGF-I) promotes the proliferation and differentiation of alveolar epithelial cells during the repair of lung injury [76]. In insulin resistance and T2DM, with or without obesity, defective IGF-I was reported [77], rationalizing the poor prognosis of COVID-19 when lung injury becomes irreparable. Furthermore, defective baseline ACE2 in diabetics [78], possibly exacerbated by the virus-mediated ACE2 downregulation, aggravates the viral inflammatory response with disequilibrated injurious ACE/ANGII/AT1 against low ACE2/angiotensin7-9/AT2 and Mas [39].

Clinical data highlighted the link between circulating TNF-α on one side and insulin resistance and T2DM on the other side [79]. However, this link could be reliable at the onset of the disease but might not apply to DM progression [80]. Anecdotal reports suggested that, in presence of hyperglycemia, the expression of TACE is increased, which favors the shedding of ACE2, and its re-distribution to the lungs [81], possibly avoided with TACE suppression, which was claimed to improve the obesity-induced insulin resistance state as mice models would suggest [82].

Neurological symptoms of COVID-19

According to the CDC [83] and Mayo Clinic [84], COVID-19 symptoms may appear two to 14 days after viral exposure and can range from mild to severe symptoms, including, but not limited to, fever, chills, cough, sore throat, fatigue, dyspnea, muscle or body aches, headache, diarrhea, nausea and vomiting, runny nose, and loss of taste or smell. Although the most likely cause of death, especially at older ages, is respiratory failure, respiratory symptoms might be milder relative to severe radiological findings [85]. The droplet infection flares up in vulnerable groups to severe pulmonary inflammation, pneumonia, ALI, ARDS, and sepsis culminating in multi-organ failure, mainly respiratory, in addition to cardiac or renal failure [86,87].

When accessing the brain, the virus elicited acute neurological complications, among them are viral or toxic encephalopathy, myelitis, meningoencephalitis, confusion, seizures, stroke, ataxia, agitation, delirium, insomnia, depression, ageusia, and anosmia [88]. Furthermore, the virus can locate itself in the brain to provoke cognitive neuropsychiatric disorders, more commonly in those with predisposing neurodegenerative and neuro-inflammatory diseases. The severity of the infection, along with comorbidities, was associated with a higher incidence of neurological symptoms. Neurological affection by the virus and its inflammatory responses can yield long-term sequelae, such as cognitive impairment, memory deficits, and defects in attention and execution. The virus may remain dormant in the nervous tissue and immune cells, manifesting later as a neuropsychiatric illness [89]. This was observed in the recovery period during the past outbreaks when patients infected with SARS or MERS were afflicted with PTSD, depression, obsessive-compulsive disorder (OCD), panic disorders, anxiety, irritability, memory impairment, fatigue, and insomnia [90]. Similarly, in the current post-COVID-19 recovery period, patients are experiencing sleep problems, cognitive issues, fatigue, and memory defects. The cognitive decline noticed during the post-COVID-19 recovery period can follow hospital discharge as late as four weeks [91], owing to psychiatric issues deterring adult neurogenesis [92], and culminating in cognitive dysfunctions [93].

The wide expression of ACE2 in the brain justifies the viral brain infiltration. The blood-brain barrier at the olfactory bulb (OB) is more permeable than at other brain regions [94] with ACE2 expression in the blood vessels lining the olfactory mucosa facilitating the entry of SARS-CoV-2 through the OB [95]. Other than the olfactory route, the gut-brain axis is another way where the virus reaches the brain through enteric nerves [96]. Systemic dissemination from the epithelial airways is a third way of brain infection [97]. Additionally, the activated viral-infected leukocytes can readily access the blood-brain barrier [98]. Once in the brain, SARS-CoV-2 attaches to ACE2, with subsequent release of neuro-inflammatory mediators [99]. Regardless of the loophole that allowed central viral invasion, once inside, the virus seems to lock the door behind and bury its portal of entry by downregulating ACE2 [100], leaving the brain deprived of the neuroprotective ACE2 activity, mimicking what was demonstrated in a mouse model of Alzheimer’s disease (AD) [101]. The upsurge of pro-inflammatory cytokines including IL-6, IL-1β, and IFN-α, is also a crucial factor in suppressing adult neurogenesis [102].

During the active disease stage, anosmia seemed to occur, not only secondary to defective adult neurogenesis of OB [103], associated with reduced grey matter volume [104], but also as an attribute to functional compensation and enhanced neurogenesis [105]. Due to the late-onset of memory, linguistic and executive issues correlated with prolonged olfactory dysfunction three to six months after COVID-19 infection, rather than the severity of the infection [106]. The olfactory dysfunction course can signify an impending neurocognitive disorder [107] as odor perception, discrimination, and identification involve multiple cortical areas [108]. This is a bothersome issue since many COVID-19 cases experienced prolonged anosmia. We would expect a future increase in neuropsychiatric and neurocognitive disorders that cannot be predicted at the time.

To date, OXs neuropeptides were proposed as potential candidates for olfactory neuromodulation between the fed/fasting states [109]. The brain distribution of OXs extended to areas involved in odor processing [110]. Knowing that OXs and their receptors are also found at peripheral olfactory sites [111], a study indicated their salient role in the sense of smell. Other than the brain and peripheral olfactory sites, OXs have been detected in the enteric nervous system (ENS), especially enriched in the duodenum, and are highly expressed during fasting. OX receptors (OXRs) were found in rats, mice, guinea pigs, and humans [112].

Orexins (hypocretins)

OXs, orexin A (OXA) and orexin B (OXB), are produced from a single polypeptide, pre-pro-orexin [113]. The sequence of pre-pro-orexin in humans and mice is 83% and 95%, respectively, identical to that of rats [114]. OXs show structural homology with the glucagon/vasoactive intestinal peptide (VIP)/secretin family peptides [115]. OXA and OXB are neuropeptides, involved in wakefulness, cognition, mood, energy metabolism, analgesia, gastric acid secretion, gastrointestinal (GI) motility, blood pressure, heart rate, respiratory rate, and tidal volume [116]. Impaired orexigenic signaling was associated with narcolepsy in humans and canines, which was modeled in mice by ablation of orexigenic neurons.

As the hypothalamus was the first identified site of secretion, they were named "hypocretins," while the nomenclature "orexins" is derived from the Greek word "orexis," which means "appetite" [117], owing to their orexigenic "appetite-stimulating" effect. Their structures were deemed similar in humans and mice and their gene structure is conserved among vertebrates [118]. Experimental work attests to almost similar brain patterns of OXA in rats and mice [119]. The amino-acid sequence of OXA in humans and rodents is also identical to that of cows [120], which is not the case for OXB, in which the amino-acid sequence is similar in rats and mice, but different from humans [118]. During the postnatal period, the brain orexigenic neurons developed late in both mice and rats [119,121]. This was opposed by a study indicating their early development in rats [122]. The orexigenic neurons are excitatory ones, depicting the vesicular glutamate transporter 2 (VGLUT2) [123], producing dynorphins, glutamate, and nitric oxide [124], among other neurotransmitters. Moreover, these neurons can increase the inhibitory neurotransmitters [125].

**Figure 2:**
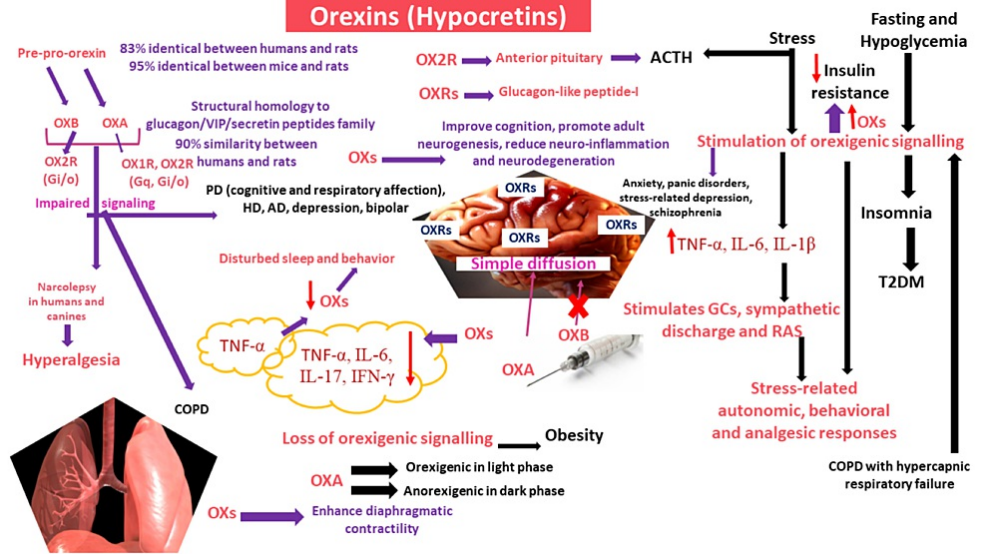
The orexigenic system: involvement in neurological disorders, obesity, diabetes mellitus, stress, and inflammation. The proteolysis of pre-pro-orexin yields OXA and OXB, both of which bind to OX2R, while only OXA binds to OX1R. Impaired orexigenic signaling is implicated in narcolepsy, and cognitive and respiratory dysfunctions in PD, HD, AD, and depression, while overactivation of the orexigenic system was found in anxiety and panic states, schizophrenia, as well as stress-related depression. OXRs, abundant in the brain, were detected in proximity to glucagon-like peptide neurons, and overactivation of the orexigenic system results in insomnia, increasing the incidence of T2DM. Conversely, higher OXs level reduces insulin resistance. The loss of orexigenic signaling in obesity and stimulation of the orexigenic signaling during fasting and hypoglycemia attest to the positive correlation between reduced OXs and T2DM. Stress was associated with stimulation of the orexigenic system, with subsequent increase in inflammatory mediators, causing activation of GCs release, sympathetic and RAS systems. In contrast, OXs can reduce inflammatory cytokines, which, in turn, suppress OXs, leading to disturbed sleep and behavior. While impaired orexigenic signaling can lead to hyperalgesia, OXs were implicated in stress-related analgesia. OXs enhance diaphragmatic contractility; however, stimulation of the orexigenic system was detected in COPD with hypercapnic respiratory failure. OXA: orexin A; OXB: orexin B; OX1R: orexin receptor 1; OX2R: orexin receptor 2; OXs: orexins; ACTH: adrenocorticotropin releasing hormone; GCs: glucocorticoids; T2DM: type 2 diabetes mellitus; PD: Parkinson’s disease: HD: Huntington’s disease; AD: Alzheimer’s disease; COPD: chronic obstructive pulmonary disease; TNF-α: tumor necrosis factor-alpha; IFN-γ: interferon-gamma; IL-1β: interleukin-1beta; IL-6: interleukin-6; IL-17: interleukin-17.

OXs bind to the G protein-coupled orexin or hypocretin receptors types 1 and 2 (OX1R or HCRTR1 and OX2R or HCRTR2, respectively), the activation of which results in altering signaling pathways linked to Gq/11 for OX1R and OX2R, Gi/o for OX2R [126], and Gs at high concentrations, resulting in the modification of calcium influx and adenyl cyclase activity. OXRs are conserved among mammals, with more than 90% similarity between humans and rats [118]. While OXA binds OX1R, both OXA and OXB bind OX2R (Figure 2) [127].

OXs, ACE2, Inflammation, and Sepsis

Despite the high expression of both ACE2 and TMPRSS2 in the small intestine, germ cells, kidneys, and heart, against the medium level of expression in the lung tissue and low-to-medium expression in the nervous system [21], COVID-19 symptoms did not reflect such differential ACE2 expression, instead, the respiratory symptoms affected most of the cases, and neurological symptoms were found in 30% of hospitalized cases [128], while diarrhea, for instance, was found in a smaller number of cases [129]. The neurological symptoms might be related, in some cases, to the GI affection, as SARS-CoV-2-mediated shedding of enteric ACE2 [130] might have altered intestinal microbiota, together with reduced antimicrobial peptides, as occurred with ACE2 knockout animals [131]. Such disturbed enteric microbiome was linked to defective cognition and adult neurogenesis [132], leading to psychiatric issues, such as anxiety and depression [133]. This justification could be authenticated if neurological affection was associated with or preceded by GI disturbance, but what if the main patient’s complaint was related to neurological issues during the active stage or in the post-COVID-19 recovery period? Perhaps, other pathways should be explored to unveil such discrepancy.

In fact, the orexigenic system and the RAS system, specifically ANGII and ACE2, share variable functions (Table 1).

**Table 1:** Comparison between functions of the renin-angiotensin system and orexigenic system. ANGII: angiotensin II; ACE: angiotensin-converting enzyme; ACE2: angiotensin-converting enzyme 2; AT1R: angiotensin receptor 1; AT2R: angiotensin receptor 2; HPA: hypothalamic-pituitary-adrenal; TACE: tumor necrosis factor/angiotensin convertase; OXs: orexins; ANG (1-7): angiotensin (1-7); OXA: orexin A; OXB: orexin B; ARDS: acute respiratory distress syndrome; OXRs: orexin receptors; T2DM: type 2 diabetes mellitus; GCs: glucocorticoids; CRH: corticotropin-releasing hormone; RAS: renin-angiotensin system.

Items of comparison	Renin-angiotensin system (RAS)	The orexigenic system
Orexigenic activity	ANGII	OXA
Pro-apoptotic	ANGII	OXs
Adult neurogenesis	ACE2	OXs
Counteract endothelial cell dysfunction	ANG (1-7)	OXA
Inflammatory bowel disease	Exacerbation with ACE2 deficiency	Anti-inflammatory effects of OXA and OXB
Rheumatoid arthritis	ACE2 upregulation	Anti-inflammatory effect of OXA
Septic shock	ANGII targeted the cardiovascular disturbances	Ameliorated using systemic OXA administration
	Exacerbation of ARDS with ANGII/AT1R upregulation	
	Alleviation of ARDS through ACE2/AT2R pathway	
Obesity	Compensatory protection by ACE2 upregulation	Loss of signaling
	Higher pulmonary ACE2 expression	Low plasma OXA
Diabetes mellitus	Defective baseline ACE2	Reduced insulin resistance with elevated OXs
	Higher pulmonary ACE2 expression	Insulin stimulates OXRs
	Enhanced TACE activity and tissue ACE2 shedding	Stimulation of pancreatic insulin secretion by OXRs is blocked by OXA antagonists
	TACE suppression enhances obesity-induced insulin resistance	Increased T2DM risk with insomnia and increased OXs
Stress and HPA axis	GCs enhance ACE activity in the lungs	OXs stimulate adrenal GCs
	ANGII triggers stress-related behaviors	Immobilization stress enhanced brain OXRs expression
	ACE2 overexpression reduced plasma corticosterone response to stress	OXA increased brain CRH and blood corticosterone
		Mediate the stress-related autonomic and behavioral responses
		Activate RAS
Analgesia	ANGII	OXA

In vitro studies highlighted the appetite-stimulating activity of both OXA and ANGII [134]. OXs were described as pro-apoptotic mediators [135], an effect that was also ascribed to ANGII when applied to neuronal cell culture from neonatal rat brains [136]. OXs and ACE2 were also implicated in the proliferative phase during adult neurogenesis [137]. The cerebral endothelial dysfunction was counteracted by ANG (1-7), a byproduct of ACE2 [138], and OXA was able to suppress endothelial cell inflammation and dysfunction as well [139]. Moreover, both OXs and ACE2 could benefit inflammatory disorders, as inflammatory bowel disease was exacerbated in ACE2 deficiency states [131], and, in ulcerative colitis, OX1R was upregulated in the colonic mucosa, mediating the anti-inflammatory effects of both OXA and OXB [140]. In patients with rheumatoid arthritis, ACE2 was upregulated in fibroblast-like synoviocytes (FLSs), owing to IL-6 stimulation [141], possibly, conferring a compensatory anti-inflammatory activity. When modeling rheumatoid arthritis, TNF-α administration reduced OXA in FLSs. In contrast, when OXA was given to TNF-α-treated FLSs, it exerted an anti-inflammatory effect, reducing IL-1β, IL-6, and IL-8, and suppressing the TNF-α-mediated activation of NF-κB [142]. This applied to central inflammation as challenging mice with TNF-α reduced the hypothalamic OXs expression, impacting sleep and behavior [143].

Regarding sepsis, a lethal complication of bacterial, viral, fungal, or parasitic infections [144] and inflammatory and coagulation responses was responsible for the multi-organ dysfunction syndrome [145], leading to an upstroke of inflammatory cytokines, including TNF-α, IL-6, and IL-1β, along with others [146]. In experimentally induced septic shock, ANGII was demonstrated to benefit the cardiovascular disturbed dynamics [147]; however, in mice model of sepsis-induced ARDS, the condition was aggravated in presence of upregulated ANGII/AT1R, but the pulmonary injury was defeated through ACE2/AT2R pathway [148]. Like ACE2, OXs appear to reduce the deleterious effects of septic shock when in a mouse sepsis model, OXs intracerebral administration regulated blood temperature and heart rate, with increased adrenocorticotrophic hormone (ACTH) level [149]. In another mouse model of endotoxic shock, the systemic administration of OXA was able to access the brain and reduced TNF-α, IL-6, IL-17, and IFN-γ during systemic inflammation, with subsequently improved survival [150].

Orexins in Obesity and Diabetes Mellitus

Loss of orexigenic signaling was linked to obesity [151]. OXA was lower in obese when compared to lean individuals. The higher the BMI, the lower the plasma level of OXA [152]. The orexigenic neurons contain leptin, a peptide secreted in proportion to the amount of fat stored in adipocytes, and its exogenous administration reduces weight [153]. Presumably, OX1R is more involved than OX2R since fasting and leptin increased and decreased, respectively, the expression of OX1R, but not OX2R [154].

In experimental settings, OXA delayed rat’s grooming, a satiety behavior when transitioning from eating to resting [155]. Interestingly, in adult rats, the orexigenic activity of OXA followed a circadian pattern, when the intracerebroventricular (icv) injection of OXA stimulated appetite during the light phase, versus an anorexigenic activity during the dark phase [156]. Increased wakefulness as a response to fasting might also be mediated by stimulation of the orexigenic systems [157]. Surprisingly, in mice, the ablation of orexigenic neurons resulted in hypophagia, but late-onset obesity was noticed [158]. Notably, the activity of OXRs was lacking in chickens [159], rhesus monkeys [160], and old rats [161], and operated differently in sheep [162].

In several brain areas, including the OB, glucose-sensing neurons (GSNs) [163] respond to extracellular glucose levels. The optimum functioning of such GSNs can prevent obesity, T2DM, and neurodegenerative diseases [164]. The brain also receives vagal afferents from glucoreceptors in the gut and liver that relay information to an area with abundant orexigenic neurons [165]. OXs might be related to the brain glucose requirements, with subsequent effects on the processing of sensory inputs [166]. Hypoglycemia was found to activate the orexigenic system and increase central OXB level [167]. Both hypoglycemia and OXA stimulate the GSNs [168]. Both OX1R and OX2R exist in pancreatic β cells, while OXA was found in α cells [169]. OXR are synthesized outside the pancreas [165].

As defective baseline ACE2 was implicated in T2DM, evidence is mounting regarding similar OXs change. The higher the peripheral OXs concentration, the lower the insulin resistance in patients with T2DM [170]. Insulin was able to stimulate OXRs, in turn, OXA injection provoked pancreatic stimulation following insulin-induced hypoglycemia, an effect that was blocked using an OXA antagonist [171]. In the presence of glucose, OXRs stimulated in vitro pancreatic insulin secretion [172]. Since the activation of OX induces insomnia, and the risk of T2DM was increased with disturbed sleep and increased OX, OX antagonists improved glucose tolerance when administered to mice models of T2DM during their resting phase [173]. Conversely, increased appetite in rats due to either insulin-deficient diabetes or palatable foods was not associated with increased brain levels of pre-pro-orexin mRNA [174], outlining that not all hunger stimuli activate the orexigenic system.

In vivo studies discriminated between OXA, which increased both blood glucose and insulin, and OXB, which exclusively increased blood insulin levels without affecting blood glucose [175]. OXA inhibited GSNs in the brain, which are stimulated by glucose, conveying a satiety feeling. Although debatable, in fasted rats, continuous high-dose OXA increased plasma glucagon and plasma glucose, while reducing plasma insulin. In other studies, OXA did not affect glucose or insulin levels and rather decreased plasma glucagon [176]. Dietary changes modify the response of brain orexigenic neurons, which is stimulated with high lipids, low glucose, and fasting, while inhibited when lipids are low and glucose is high [177].

Orexins and the COVID-19 Pandemic Stress

The drastic social and economic changes associated with the COVID-19 pandemic, including the lockdown, social isolation, and studying and working from home, added to reduced salary and availability of food, the crowded health facilities, and lack of sufficient medical personnel and resources, and such stressors flood caused flaring up of mental health disorders [178]. The stress itself can increase the likelihood of COVID-19 morbidity [179], as stress can reduce immune responses to infections, and even vaccination [180]. The involvement of mast cells, abundant in the lungs, brain, and GI tract, is shared between viral infections and psychological stress [181]. The release of inflammatory mediators from mast cells due to stress is one factor that can trigger neuroinflammatory diseases such as AD and traumatic brain injury (TBI) [182].

Stress, among other extrinsic factors, can negatively modulate adult neurogenesis [183]. The stressful pandemic situation activated the hypothalamic-pituitary-adrenal (HPA) axis, starting with the release of the corticotropin-releasing hormone (CRH) with subsequent increased adrenal glucocorticoids (GCs), as an anti-stress hormone [184]. GCs were known to exacerbate viral infections during psychologic stress periods [185], up to causing the defective response to vaccination [186], owing to impaired CD8+ T cells-viral antigen presentation [187], as shown during the early stages of influenza viral infection [188], with further progression to hospital-acquired pneumonia [189]; unlike community-acquired pneumonia, which benefited from GCs administration [190].

Although evidence is scarce and inconclusive about the selective effects of GCs on ACE2, especially at the pulmonary level, yet GCs potentiated ACE activity in endothelial cells and normal rat lungs [191]. As a known neuropeptide mediator of stress responses, ANGII, by binding to AT1R in the brain, can trigger stress-related behaviors [192,193]. GCs have long been identified as enhancers of AT1R and their gene expression [194]. In mice overexpressing ACE2, both reduced plasma corticosterone and lower corticosterone response to restraint stress were noticed. Knowing that ACE2 can reduce stress responses by suppressing CRH [118], we can speculate that SARS-CoV-2-induced internalization of ACE2 could abolish such an anti-stress effect [195] and that COVID-19 amplifies the stress-linked behavior.

Differential stress response was correlated to gender-specific variation, with females having a more effective orexin system and hyperresponsiveness to stress, culminating in psychological disturbances such as major depression and PTSD [196]. Regulatory effects of OXs over the pituitary gland might be responsible for the gender-specific differences between males and females [197]. The anterior pituitary contains OX2R, localized on basophil cells, which release ACTH [198]. The protein expression of pituitary OX1R and adrenal gland OX2R was higher in males than females [199]. In males, adrenal OX2R was even higher than in the brain. Differential adrenal distribution was characterized for OXs receptors, with OX1R localized in the adrenal cortex and OX2R in the adrenal medulla [200]. By acting on adrenal OX1R, OXs stimulate adrenal glucocorticoid secretion [201].

Multiple stressors were able to provoke the orexigenic system, such as immobilization, cold exposure, conditioned fear, and food shortage [202]. Following immobilization stress, the OXRs protein expression was increased in the brain. The injection of OXA into the brain elevated blood corticosterone, CRH, and arginine vasopressin [203], which were blocked using neuropeptide Y antagonists [204]. Such an OXA-mediated effect was relevant when dealing with adrenal corticosteroids in both humans and rats and mostly mediated through OX1R [201]. Presumably, the orexigenic neurons mediate the autonomic and behavioral responses when afflicted by emotional or fearful stimuli, as evidenced by reduced cardiovascular and behavioral responses in the resident-intruder paradigm in pre-pro-orexin knocked out mice [205] and stimulation of grooming and chewing of inedible material, mediating the delayed increase in the amount of rapid eye movement (REM) sleep [206], along with the activation of the monoamine system [207]. The stress-induced responses in rats were obliterated using an OX1R antagonist or RNA interference to orexin [113]. However, other stress responses were not attributed to OXA as thermogenesis and cardiovascular response to cold exposure [208].

Psychological stress can trigger psycho-somatic manifestations including GI symptoms, shortness of breath, headache, insomnia, and cardiovascular manifestations [209]. OXs might contribute to the hypertensive response to stress [113], which was abolished using almorexant, a dual OX1R and OX2R antagonist [210]. The orexigenic system was reported to activate the sympathetic nervous system [211,212], probably by acting centrally on brain adrenergic neurons [213] or through preganglionic sympathetic nerve fibers [214] and the peripheral RAS system, so that almorexant can antagonize RAS, secondary to reduced renal sympathetic activity and subsequent renal renin release [210]. Besides, OXA stimulates sympathetic activity by enhancing TNF-α and IL-6, IL-1β, and Fos-related antigen 1 [215]. The stress-induced responses such as analgesia, ACTH elevation, and cardiovascular changes, mediated via OXA can be inhibited using OX2R antagonist as well [216]. In turn, the OX system coordinates with the nociception/orphanin FQ systems to modulate stress-induced analgesia (SIA) [217,218], so that pre-pro-orexin knocked out mice exhibited hyperalgesia and less SIA [219]. Such antinociceptive activity is supported by the increased prevalence of chronic pain in patients with narcolepsy linked to defective OX signaling [220]. If stress, ANGII [221], and OXs provide analgesia [222], at least OXs will reduce the stress-related neuropsychiatric issues and the ANGII-linked cardiovascular stress responses.

The orexigenic system and neurological disorders

In the brain, OXs-producing cells were detected in frogs, rodents, hamsters, guinea pigs, bovines, and humans [223]. OXs nerve fibers ramify, extensively, to be found, abundantly, in the cerebral cortex, OB, brain stem, and spinal cord, among other brain regions [126]. Unlike OXB, OXA penetrates the blood-brain barrier by simple diffusion [224].

The pre-pro-orexin gene was proposed as a candidate for neurodegenerative diseases collectively called “chromosome 17-linked dementia,” referring to the location of the gene in chromosome 17q21 [225]. A growing body of evidence points to the involvement of the orexigenic system in Parkinson’s disease (PD), Huntington’s disease (HD), and AD [226]. In PD, the loss of orexigenic neurons [227,228] has been correlated to disease progression [229] with an eventual reduction in cerebrospinal fluid (CSF) OXA [230]. In mice, such loss was more evident late in the disease stage [231]. However, the loss of orexigenic neurons in PD was attributed to reduced OXs, rather than neuronal death [226]. The defective orexigenic system in PD might be responsible for the associated cognitive decline and the respiratory dysfunction detected in mice showing reduced respiratory response to hypercapnia [232]. The orexigenic system obliteration found in PD-associated respiratory affection was also detectable in patients with chronic obstructive pulmonary disease (COPD) depicting reduced plasma OXA [233]. In contrast, COPD patients with hypercapnic respiratory failure exhibited high plasma OXA [234].

In post-mortem specimens of patients with advanced AD, the amount of orexigenic neurons and the OXs level in the CSF were reduced [235]. Conversely, the age-related reduction of quality of sleep was demonstrated to increase, both OXA in CSF and the pathognomonic tau protein of AD [236]. During aging, the OX1R expression was lower than at younger age, and the amount of orexigenic neurons in the brain was either lost [237] or preserved [238], while plasma and CSF OXA was either low [239] or high [240]. Recently, OXA was identified as a neuroprotective factor [241,242], reducing neuronal loss, neurodegeneration, and neuroinflammation.

OXs might enhance cognition, by inducing neuronal excitability in the prefrontal cortex, through pre-synaptic and post-synaptic pathways [243,244]. Despite the fact that OXA was linked to impaired rats’ performance in the Morris water maze test and suppressed long-term potentiation (LTP) [245], experimental work in rats and primates proved that both OXA and OXB have a certain role in improving cognition, using the icv, IV, or intranasal routes. In patients with narcolepsy, the ability to make decisions was affected [246]; hence, OXs were explored as cognitive enhancers [247] to ameliorate the insomnia-deteriorated cognitive performance [248], by promoting working memory, as demonstrated in the active and passive avoidance settings [249]. These favorable OXs effects over cognition were antagonized by OX1R blockers and OXs neuronal lesions [250,251]. Nevertheless, other studies negated the relationship between orexin antagonists and cognitive defects [252].

Advantageously, in an experimental model of depression, developed using a dual orexin receptor antagonist (DORA), the analysis revealed the antidepressant capability of OXs [253]. In line with such findings, patients with bipolar exhibited low plasma OXA [254]. Conversely, in anxiety and panic disorders, rodent models identified the involvement of OXs [255,256], and patients with panic disorders had elevated OXA [257]. As for schizophrenia, human studies were conflicting; a study involving female patients noticed the hyperactivity of the orexigenic system [197], another study emphasized the positive link between OXA level and negative symptoms of schizophrenia, including cognitive impairment [258], while a recent meta-analysis and systematic review did not find any relevant change [259].

Other than the neurocognitive and neuropsychiatric disorders, central OXA administration appears to present novel centrally acting non-opioid analgesics, since their effects are not blocked by opioid antagonists, but rather abolished using adenosine antagonists [260]. Unfortunately, systemic injection of OXA did not replicate such anti-nociception activity [261].

The thermoregulatory brain areas, including those involved in temperature regulation, heat loss responses, and shivering, express orexigenic neurons, which, along with OXs, can release other co-transmitters such as glutamate, dynorphin, and nitric oxide [262], postulating that this system has some therapeutic role as antipyretic.

The future: orexin agonists versus antagonists

Despite the fact that OXs seem to benefit respiratory problems, being involved in the hypercapnic chemoreflex response and the phrenic and ventilatory long-term facilitation [263], along with facilitation of upper airway patency [264], together with the central administration increasing tidal volume and minute ventilation [265], added to enhance the diaphragmatic contractility through triggering phrenic nerve firing [266], these effects might be relevant to mice, but not humans [267]. OX antagonists might offer promising medications in the case of sleep apnea, knowing that patients with sleep apnea syndrome exhibited elevated OXA [268] and that hypercapnia stimulates the orexigenic neurons, with sympathetic facilitatory activity, precipitating hypertension during the night.

OX antagonists can block both OX1R and OX2R, so called DORAs, or can block one single receptor subtype, hence called single orexin receptor antagonist (SORA). Considering the common mechanism of action of conventional hypnotics, reinforcing gamma-aminobutyric acid (GABA) neurotransmission, precipitating daytime sedation, and motor instability and incoordination, added to their abuse because of tolerance and dependence [269], OX antagonists seem privileged, to some extent. Most of the research work converged on DORAs, of which some members received the FDA-approval, promoted as sleep aids, by their novel mechanism involving the reversible binding to OXRs, supporting the induction and maintenance of sleep [270,271]; however, they can lead to daytime sleepiness, suicidal ideation, and respiratory depression [272].

As for OX agonists treating narcolepsy, they were shown to ameliorate cognition in AD and PD, based on evidence that OX deficiency reduces spatial working memory. This remains controversial as reduced orexigenic signaling in patients with narcolepsy was associated with decreased beta-amyloid and neurodegeneration of AD [273], eventually encouraging the recent use of DORAs for the treatment of insomnia in mild-to-moderate AD patients [274].

DORAs are promising future therapeutic modalities in psychiatric disorders, such as in panic and anxiety states [275]. DORAs are of special value with co-existing schizophrenia or PD [276]. Despite low OXs levels during depression, orexin antagonists showed some success in treating depression by modulating stress responses [277]. In this setting, they share similar effects with ACE inhibitors [278], activating the ACE2 pathway as linked to anxiolytic and antidepressant activities [279].

Both SORAs and DORAs disclosed the involvement of the orexinergic system in epilepsy [280], migraine, and cluster headache [281]; therefore, subjected to ongoing clinical trials. Given the potential cardio-protection of OX2R agonists [282], selective OX1R might be favored in patients with cardiac comorbidities.

## Conclusions

Since 2019, the clinical course of COVID-19 revealed neurological affection during the active disease stage and the post-COVID-19 recovery period. Special consideration was given to high-risk groups, such as patients with obesity and DM, susceptible to COVID-19 morbidity and neurological derangements. The stress precipitated by the recurring COVID-19 waves, with the associated socio-economic burden, has further exacerbated the neurocognitive and neuropsychiatric disorders while increasing the vulnerability to COVID-19 morbidity by suppressing the immune system and increasing pro-inflammatory cytokines. Special interest has been directed toward ACE2 as the portal of entry of SARS-CoV-2, along with the lethal cytokine storm, both of which provoked multiple neurodegenerative and neuro-inflammatory disorders. Yet the pattern of differential distribution of ACE2 between tissues does not match the clinical course and consequences of the infection, showing lower brain and pulmonary expression against its abundance in gastrointestinal and renal tissues.

Multiple functional similarities between ACE2 and OXs exist in terms of altered expression in obesity and DM, ameliorating the stress-related responses, having anti-inflammatory profiles, and enhancing neuronal survival. The distribution of the orexigenic system along the olfactory and enteric routes of SARS-CoV-2 invasion to the brain makes it a potential tool exploited by the virus, but also a prospective pharmacologic target. The presumptive analgesic and antipyretic activities of OXs might add to their privileged pharmacodynamics. Based on animal and human studies emphasizing the role of OXs in neurological disorders, whether defective or upregulated, and as pharmacologic manipulation with available agonists and antagonists is possible, elaborative work should be done to determine the role of the orexigenic system in COVID-19, if any, with special care to the neurological, respiratory, and cardiovascular aspects. Further pre-clinical and clinical studies are recommended to identify and verify whether activating or blocking the orexigenic system would help limit the disease morbidity and mortality.
